# Prevalence and knowledge of gender dysphoria among young medical trainees

**DOI:** 10.1192/j.eurpsy.2025.2314

**Published:** 2025-08-26

**Authors:** N. Regaieg, F. Guermazi, D. Mnif, A. Jmal, M. Ben Jemaa, F. Cherif, I. Baati, J. Masmoudi

**Affiliations:** 1 Psychiatry A department, Hedi Chaker University Hospital; 2 Family medicine department, University of Sfax, Faculty of medicine of Sfax; 3 Community Health and Epidemiology Department, Hedi Chaker University Hospital, Sfax, Tunisia

## Abstract

**Introduction:**

Gender dysphoria (GD) is a rare entity which involves significant distress experienced by an individual due to a perceived discrepancy between his gender identity and his sex assigned at birth. In the current literature, there is little research on medical students’ knowledge and attitudes towards this entity.

**Objectives:**

The objectives of our study were to estimate the prevalence of gender dysphoria within young medical trainees and to explore their knowledge and beliefs about this entity.

**Methods:**

It was a cross-sectional and descriptive study, carried out on GOOGLE FORMS in the period of time from October 1, 2023, to January 31, 2024, and relating to a population of Tunisian young medical trainees. We used a questionnaire including an information sheet and the gender identity/gender dysphoria questionnaire for adolescents and adults (GIDYQ-AA).

**Results:**

Our study involved 111 participants with a sex ratio (M/F) of 0.56. Their median age was 28 years.

The overall prevalence of gender dysphoria was 0.9%. It was 2.5% among participants assigned male at birth (95% CI=[0.06%–13.2%]) and 0% among those assigned female at birth.

Among the participants, 21.6% had received training on GD or sexual identity disorders during their medical education while 3.6% of reported being aware of specialized services for the management and support of GD.

Four-fifths of the participants (80.2%) believed that the development of GD would be due to organic factors, while 70.3% described the contribution of socio-cultural factors and 58.6% attributed it to early family interactions.

More than a third of the participants (40.6%) considered GD as an entity related to sexual development disorders, 28.8% equated it with a symptom of a more general mental pathology, while 30.6% regarded it as a distinct entity.

Regarding the suffering associated with GD, it was attributed to the internal experience of gender incongruence by 42.3% of the participants, social stigma in 29.8% of cases, and to psychiatric pathology by 27.9% of the participants. GD was mentioned as a risk factor for suicide by 84.7% of the participants.

**Conclusions:**

Our study revealed a low prevalence of GD among young medical trainees, similar to that observed in the general population, which could be explained by reluctance and fear of stigma in our society where sexuality remains a taboo subject. The knowledge of young doctors about this issue, still insufficient, could be improved through sexuality training dedicated to specialists, as well as through the teaching of sexology during the medical education.

**Disclosure of Interest:**

None Declared

